# Supplementary material for: EnzML: multi-label prediction of enzyme classes using InterPro signatures
Source: BMC Bioinformatics. 2012 Apr 25;13:61. doi: 10.1186/1471-2105-13-61 (PMC3483700; doi:10.1186/1471-2105-13-61)
Supplement: Addtional file 5 — The Java code to format the data files, evaluate and predict. The file enzml_java_code.tar.gz contains the Java code used to format database data to ARFF and XML formats, to execute cross and train-test (jackknife) evaluations and to record evaluation results to database. More information is included in the readme.txt file and the Javadoc files. The code can be used with a MySQL database. To use a different database software, other JDBC drivers might be required. [file 1471-2105-13-61-S5.gz › java_code/enzml2011/doc/uk/ac/ed/inf/taweka/mulan/attributesfilter/AttributesFilteredArff.html]

AttributesFilteredArff


---


|  |  |  |
| --- | --- | --- |
| **Overview** \| **Package** \| **Class** \| **Use** \| **Tree** \| **Deprecated** \| **Index** \| **Help** | |  |
| **PREV CLASS**   **NEXT CLASS** | **FRAMES**    **NO FRAMES**     **All Classes** |  |
| SUMMARY: NESTED \| FIELD \| CONSTR \| METHOD | DETAIL: FIELD \| CONSTR \| METHOD |  |


---


## uk.ac.ed.inf.taweka.mulan.attributesfilter Class AttributesFilteredArff

```
java.lang.Object
  uk.ac.ed.inf.taweka.Initialised
      uk.ac.ed.inf.taweka.weka.Arff
          uk.ac.ed.inf.taweka.mulan.MulanArff
              uk.ac.ed.inf.taweka.mulan.attributesfilter.AttributesFilteredArff
```

**All Implemented Interfaces:**: Initialisable

---

``` public class AttributesFilteredArff extends MulanArff ```

---

**Field Summary**

**Fields inherited from class uk.ac.ed.inf.taweka.weka.Arff**

`ARFF_TABLE, AUTHOR, TAWEKA_NAME, VERSION, WEB_PAGE`

**Fields inherited from class uk.ac.ed.inf.taweka.Initialised**

`m_initialisationIsCorrect`


**Constructor Summary**

`AttributesFilteredArff(java.lang.String dataPropsFileName,
java.lang.String resultsPropsFileName,
java.util.TreeSet<java.lang.String> attributesNames,
AttributesFilter filterManager)`


| **Method Summary** | |
| --- | --- |
| `java.util.TreeSet<java.lang.String>` | `getAttributesNames()` |
| `AttributesFilteredDataSetManager` | `getDataSetManager()` |
| `AttributesFilter` | `getFilterManager()` |
| `void` | `setDataSetManager()` |

**Methods inherited from class uk.ac.ed.inf.taweka.mulan.MulanArff**

`getXmlPath, getXMLpathFromArffPath, main`

**Methods inherited from class uk.ac.ed.inf.taweka.weka.Arff**

`generateAndWriteArffFile, getArffPath, getArffRecordId, getDataArffProperties, getDataDbManager, getDataPropsFilePath, getDbConn, getDbReader, getResultsDbManager, loadAndGenerateArff, setArffRecordId`

**Methods inherited from class uk.ac.ed.inf.taweka.Initialised**

`initialisationIsCorrect, setInitialisationIsCorrect`

**Methods inherited from class java.lang.Object**

`equals, getClass, hashCode, notify, notifyAll, toString, wait, wait, wait`

**Constructor Detail**

### AttributesFilteredArff

```
public AttributesFilteredArff(java.lang.String dataPropsFileName,
                              java.lang.String resultsPropsFileName,
                              java.util.TreeSet<java.lang.String> attributesNames,
                              AttributesFilter filterManager)
```


**Method Detail**

### getAttributesNames

```
public java.util.TreeSet<java.lang.String> getAttributesNames()
```

---


### getDataSetManager

```
public AttributesFilteredDataSetManager getDataSetManager()
```

:   **Overrides:**: `getDataSetManager` in class `MulanArff`

---


### getFilterManager

```
public AttributesFilter getFilterManager()
```

---


### setDataSetManager

```
public void setDataSetManager()
```

:   **Overrides:**: `setDataSetManager` in class `MulanArff`


---


|  |  |  |
| --- | --- | --- |
| **Overview** \| **Package** \| **Class** \| **Use** \| **Tree** \| **Deprecated** \| **Index** \| **Help** | |  |
| **PREV CLASS**   **NEXT CLASS** | **FRAMES**    **NO FRAMES**     **All Classes** |  |
| SUMMARY: NESTED \| FIELD \| CONSTR \| METHOD | DETAIL: FIELD \| CONSTR \| METHOD |  |


---
